# A potential primary endpoint for clinical trials in glaucoma neuroprotection

**DOI:** 10.1038/s41598-023-34009-x

**Published:** 2023-05-02

**Authors:** Carlos Gustavo De Moraes, Keith J. Lane, Xiao Wang, Jeffrey M. Liebmann

**Affiliations:** 1 Bernard and Shirlee Brown Glaucoma Research Laboratory, Edward S. Harkness Eye Institute, Columbia University Irving Medical Center, 635 West 165th Street, Box 69, New York, NY 10032 USA; 2 Ora Clinical, Inc., Andover, MA USA; 3 Statistics and Data Corporation, Inc., Tempe, AZ USA

**Keywords:** Peripheral nervous system, Biomarkers, Medical research, Risk factors

## Abstract

The purpose of this retrospective, longitudinal study is to evaluate the relationship between MD slope from visual field tests collected over a short period of time (2 years) and the current United States’ Food and Drug Administration (FDA) recommended endpoints for visual field outcomes. If this correlation is strong and highly predictive, clinical trials employing MD slopes as primary endpoints could be employed in neuroprotection clinical trials with shorter duration and help expedite the development of novel IOP-independent therapies. Visual field tests of patients with or suspected glaucoma were selected from an academic institution and evaluated based on two functional progression endpoints: (A) five or more locations worsening by at least 7 dB, and (B) at least five test locations based upon the GCP algorithm. A total of 271 (57.6%) and 278 (59.1%) eyes reached Endpoints A and B, respectively during the follow up period. The median (IQR) MD slope of eyes reaching vs. not reaching Endpoint A and B were −1.19 (−2.00 to −0.41) vs. 0.36 (0.00 to 1.00) dB/year and −1.16 (−1.98 to −0.40) vs. 0.41 (0.02 to 1.03) dB/year, respectively (P < 0.001). It was found that eyes experiencing rapid 24-2 visual field MD slopes over a 2-year period were on average tenfold more likely to reach one of the FDA accepted endpoints during or soon after that period.

## Introduction

Intraocular pressure (IOP) reduction significantly slows the rate of visual field progression for all glaucoma subtypes and across the disease severity spectrum^1–6^. However, many patients continue to progress despite significant IOP reduction, and this is often attributed to either pressure-independent mechanisms of retinal ganglion cell (RGC) death, insufficient IOP reduction, or a combination of both^7,8^. Nonetheless, only IOP lowering has been proven to be a modifiable risk factor for glaucoma onset and progression^1–7^.

Given recent advances in our understanding of the mechanisms of RGC death, a variety of novel approaches for glaucoma neuroprotection are being investigated^9,10^. One main challenge for the development of these IOP-independent neuroprotective therapies is the lack of glaucoma progression endpoints that could be tested in clinical trials of relatively short duration in a glaucoma population treated with concomitant IOP-lowering therapies. Most clinical studies to date assessing glaucoma progression endpoints have included large populations followed for 4 or more years^1–5^; the cost of similar trials would be prohibitive for both neuroprotection proof-of-concept studies and for longer and larger clinical trials aiming for regulatory approval. Moreover, given the high efficacy of currently available glaucoma medications and surgical interventions (both laser and incisional) in slowing the rate of visual field progression, it would not be possible to run such studies on a placebo group of untreated patients^11^. Therefore, the likelihood of finding statistically and clinically meaningful differences between study arms in neuroprotection glaucoma trials would likely be small and would come with substantial cost.

To address these challenges, there have been two joint discussions between the National Eye Institute (NEI) of the National Institutes of Health (NIH) and the Food and Drug Administration Center for Drug Development and Research (FDA CDER) promoted by the Association for Research in Vision and Ophthalmology (ARVO) to discuss glaucoma progression endpoints^12,13^. The two functional endpoints currently accepted by the FDA and discussed during the NEI/FDA Glaucoma Clinical Trial Design and Endpoints Symposium are: (A) visual field progression defined as the between group mean difference in threshold for 5 or more pre-selected visual field locations that are statistically significant and have a difference that is at least 7 dB on more than one examination, and (B) visual field progression defined as five or more reproducible points of visual field locations with significant changes (P < 0.05) from baseline beyond the 5% probability levels for the Glaucoma-Change-Probability (GCP) analysis^12,14^. Given the high specificity of these criteria, identifying a population of glaucoma patients with a high proportion of subjects reaching such endpoints over a short study period (e.g. 2 years or less) is challenging and would require hundreds or thousands of patients per study arm. Per the committee’s discussion, an alternative glaucoma endpoint for pivotal trials should have a strong correlation and predictability of *either current or future* visual function, such as that measured with the above cited acceptable endpoints^12^.

Investigators have reported the advantages of using visual field mean deviation (MD) slopes as an endpoint in glaucoma clinical trials because studies employing trend analysis of visual field global indices would likely require a smaller sample size than event-based endpoints^15–19^, such as the ones currently acceptable by the FDA. The purpose of the present study is to evaluate the relationship between MD slope from visual field tests collected over a short period of time (2 years) and the current FDA recommended endpoints. If this correlation is strong and highly predictive, as the agency recommends, clinical trials employing MD slopes as primary endpoints could be employed in neuroprotection clinical trials with shorter duration and help expedite the development of novel IOP-independent therapies.

## Results

### Database characteristics

Reliable visual field tests from 490 eyes from 415 patients met the main inclusion and exclusion criteria for analyses. Of those, 20 eyes of 14 patients were excluded due to any suspicion of non-glaucomatous causes that may have affected changes in visual function, leaving a final database of 470 eyes of 401 patients. The median (IQR) number of visual field tests per eye was 12 (9 to 17) spanning 6.12 (4.07 to 9.03) years. A total of 271 (57.6%) and 278 (59.1%) eyes reached Endpoints A and B, respectively during the follow up period. 221 (47.0%) progressed more rapidly than −0.5 dB/year and 161 (34.2%) more rapidly than −1.0 dB/year.

**Figure 1:**
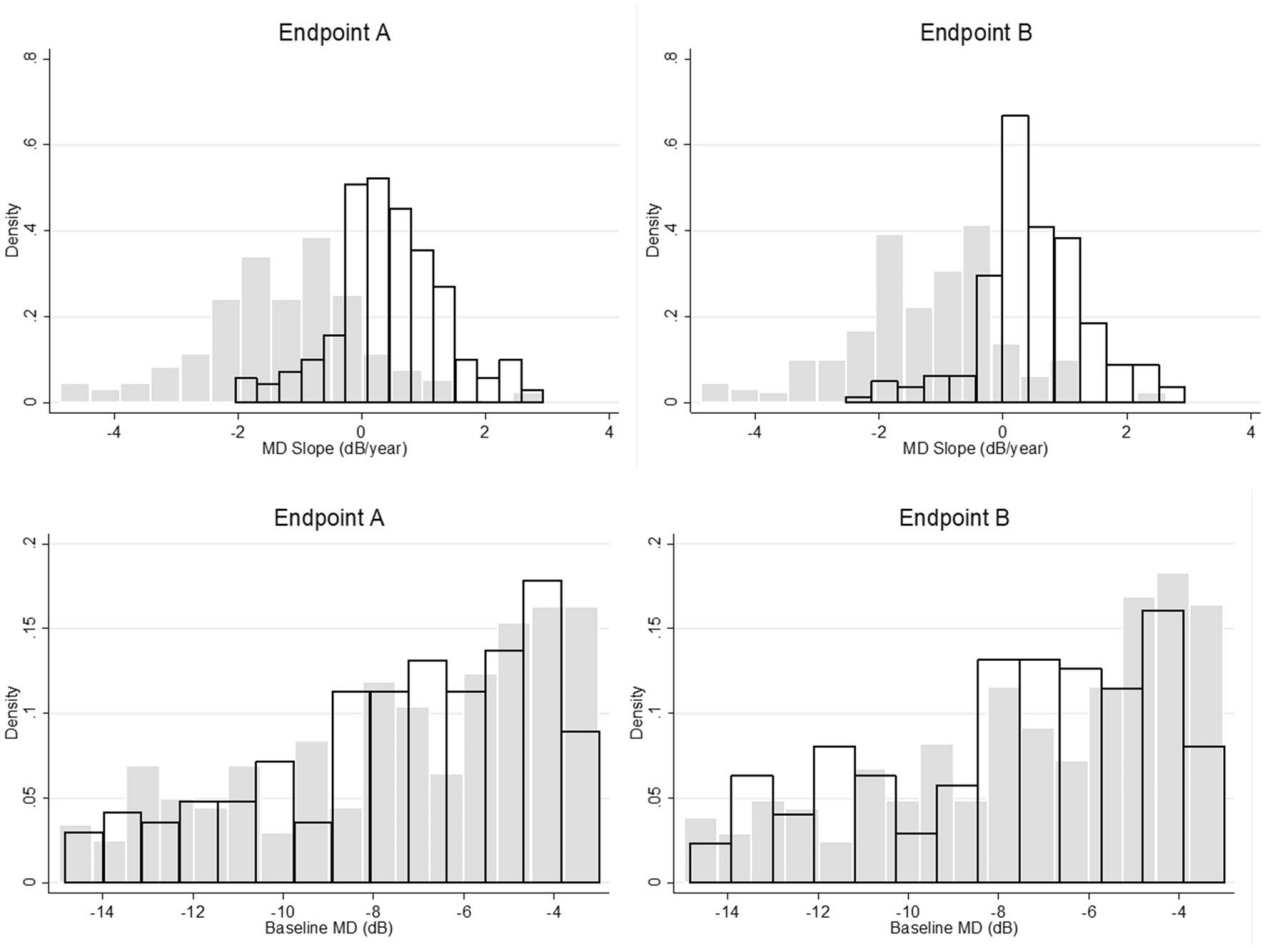
Top: distribution of mean deviation (MD) slopes by endpoint. Grey and white bars correspond to the distribution when the endpoint was met or unmet, respectively. Bottom: distribution of baseline MD by endpoint. Grey and white bars correspond to the distribution when the endpoint was met or unmet, respectively.

The median (IQR) MD slope of eyes reaching vs. not reaching Endpoint A and B were −1.19 (−2.00 to −0.41) vs. 0.36 (0.00 to 1.00) dB/year and −1.16 (−1.98 to −0.40) vs. 0.41 (0.02 to 1.03) dB/year, respectively (P < 0.001, linear mixed effects model). Clinical characteristics of the final sample are shown in Table 1. Note that the study includes a sample of patients who meet the known age demographics for established glaucoma with moderate functional damage and who performed reliable visual field tests during the study. Figure 1 depicts the distribution of MD slopes and baseline MD based upon Endpoints A and B.

**Table 1:** Patient demographics for those individuals meeting the study entry criteria.

Parameter	Median	Interquartile range
Age (years)	67.7	57.9 to 76.3
Number of visual field tests	12.0	9.0 to 17.0
Follow-up duration (years)	6.12	4.07 to 9.03
Baseline MD (dB)	−6.7	−9.5 to −4.6
MD slope (dB/year)	−0.35	−1.57 to 0.42
False-negative responses (%)	5.0	0.0 to 9.0
False-positive responses (%)	2.0	0.0 to 4.0
Fixation losses (%)	6.2	0.0 to 13.0

The values correspond to all available data from subjects included in the final analysis.

**Figure 2:**
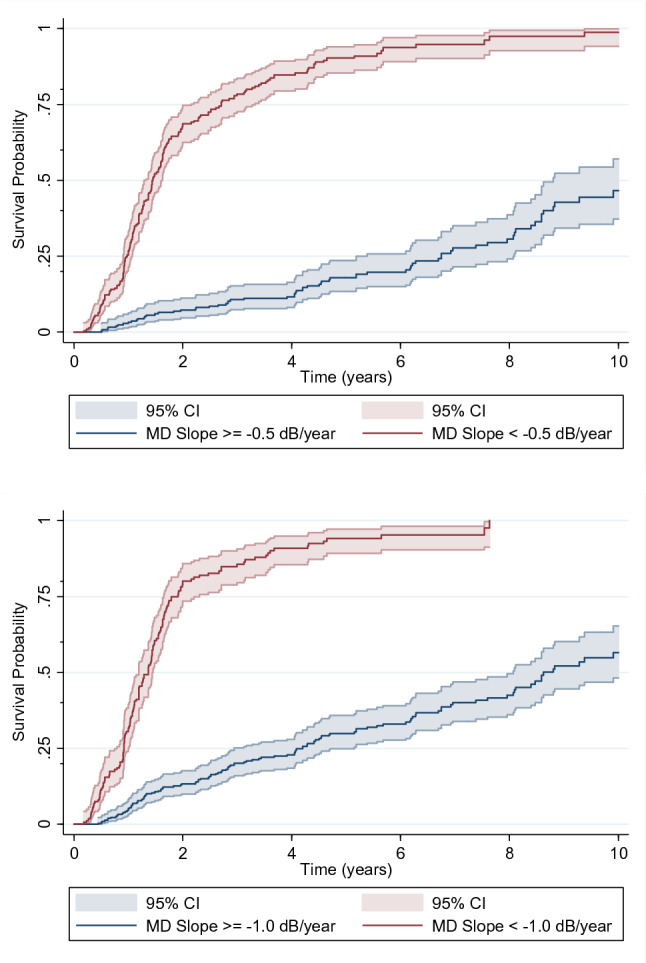
Survival curves depicting the survival probability of eyes reaching Endpoint A for different cut-off values of mean deviation (MD) slopes. Top: MD slope cut-off = −0.5 dB/year. Bottom: MD slope cut-off = −1.0 dB/year. Shaded areas correspond to the pointwise 95% confidence intervals. Time zero corresponds to when subjects were first at risk at the beginning of Sequence 1.

Figure 2 shows the Kaplan–Meier curves for Endpoint A, where the probability of progression starts been measured at Sequence 1. Each 0.1 dB/year more rapid MD slope during the first 2 years was associated with more than twofold increase in risk of experiencing Endpoint A in the subsequent years (HR: 2.17; 95% CI: 1.95 to 2.42; P < 0.001). Table 2 describes the survival times for the different cut-off values of MD slopes. Eyes with MD slopes more rapid than −0.5 dB/year were more than 10 times more likely to reach that endpoint (HR: 10.69; 95% CI: 7.72 to 14.81; P < 0.001). The overall performance of MD slopes to discriminate between eyes reaching versus not reaching Endpoint A was 87% (95% CI: 84 to 90%).

**Table 2:** Median (25th and 75th) survival times (in years) to reach Endpoint A for eyes with different pre-defined MD slopes.

Survival probability	< −0.5 dB/year	≥ −0.5 dB/year	< −1.0 dB/year	≥ −1.0 dB/year
25th	0.97	6.75	0.92	4.20
50th	1.44	10.09	1.36	8.62
75th	2.70	–	1.91	–

**Figure 3:**
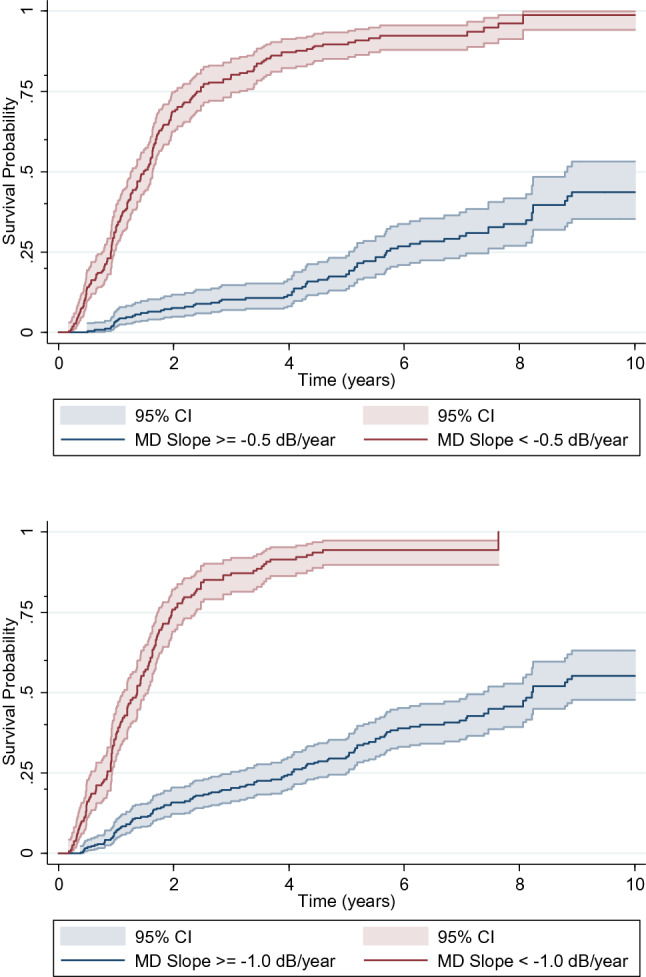
Survival curves depicting the survival probability of eyes reaching Endpoint B for different cut-off values of mean deviation (MD) slopes. Top: MD slope cut-off = −0.5 dB/year. Bottom: MD slope cut-off = −1.0 dB/year. Shaded areas correspond to the pointwise 95% confidence intervals. Time zero corresponds to when subjects were first at risk.

Figure 3 shows the Kaplan–Meier curves for Endpoint B, also where the probability of progression starts been measured at Sequence 1. Each 0.1 dB/year more rapid MD slope during the first 2 years was also associated with more than twofold increase in risk of experiencing Endpoint B in the subsequent years (HR: 2.26; 95% CI: 2.03 to 2.51; P < 0.001). Table 3 describes the survival times for the different cut-off values of MD slopes. Eyes with MD slopes more rapid than −0.5 dB/year were more than 10 times more likely to reach that endpoint (HR: 10.21; 95% CI: 7.51 to 13.88; P < 0.001). The overall performance of MD slopes to discriminate between eyes reaching versus not reaching Endpoint B was 88% (95% CI: 85 to 91%).

**Table 3:** Median (25th and 75th) survival times (in years) to reach Endpoint B for eyes with different pre-defined MD slopes.

Survival probability	< −0.5 dB/year	≥ −0.5 dB/year	< −1.0 dB/year	≥ −1.0 dB/year
25th	0.91	5.69	.85	4.05
50th	1.47	11.51	1.36	8.21
75th	2.47	–	1.96	–

**Figure 4:**
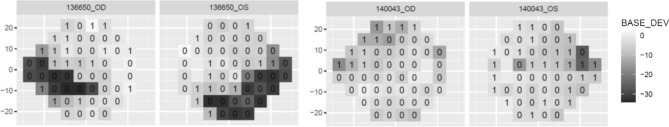
Location of progressing points as a function of baseline severity. The legend shows the relationship between baseline total deviation sensitivity (dB) and greyscale. The numbers inside each box (0 or 1) show whether that test location progressed based upon Endpoint A. Left: both eyes of the same patient who reached the endpoint. Note that there are 5 locations adjacent to a deep baseline scotoma. Right: both eyes of the same patient who reached the endpoint. Note that there are 5 locations inside a relatively shallow baseline scotoma.

**Figure 5:**
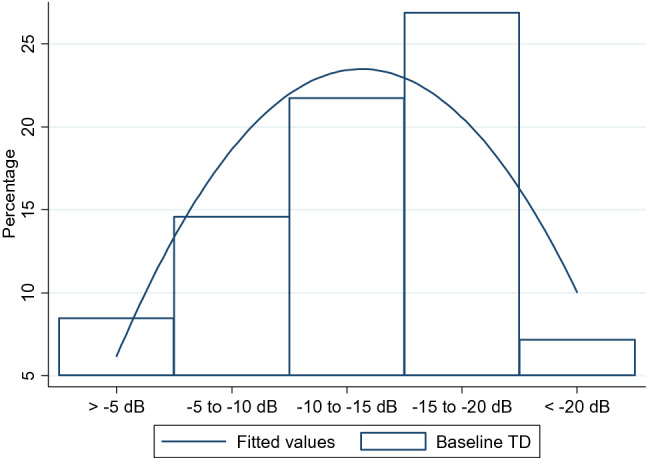
Distribution of test locations worsening by 7 dB as a function of baseline total deviation (TD) values. The fitted line corresponds to the best fit of a quadratic function.

**Figure 6:**
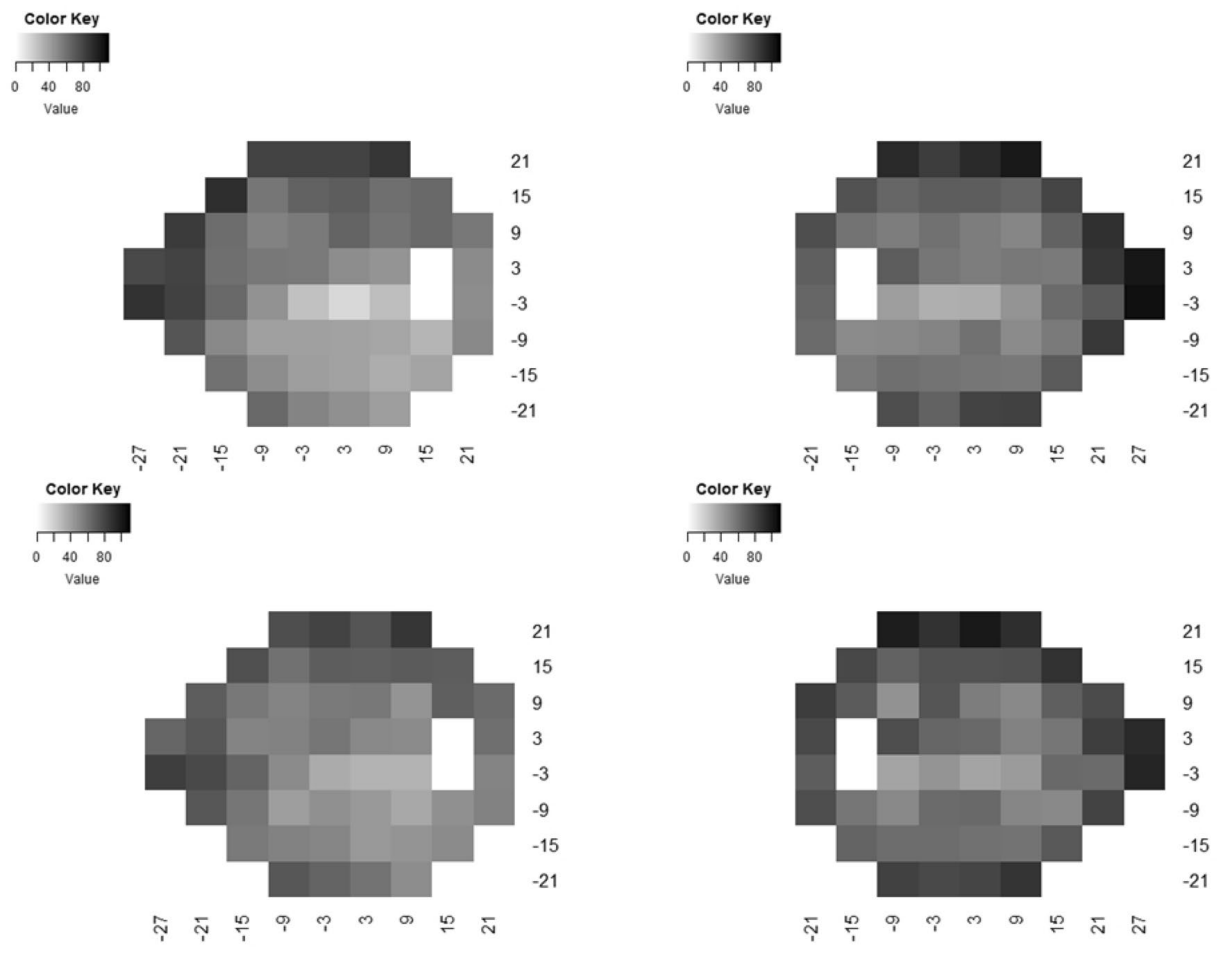
Greyscale maps depicting the frequency distribution of test locations reaching Endpoints A (top) and B (bottom) of right and left eyes for the entire final sample (N = 470 eyes). The legend corresponds to the absolute number of eyes in which a given location reached the endpoint.

To better understand the characteristics of test locations more likely to reach an endpoint during follow up, we investigated their distribution and baseline severity. Their median (IQR) baseline values were −6.5 dB (−4.0 to −11.0). We also looked at each of the 470 eyes to assess a pattern that could better describe the relationship between baseline pointwise severity and losing sensitivity according to Endpoints A and B. We found that in most cases the progressing locations (according to these endpoints) were within or adjacent to test locations with significant baseline sensitivity loss (defined as TD values < −10 dB, Fig. 4 depicts some examples). Figure 5 shows the distribution of progressing locations as a function of baseline TD values. Note that test locations between −10 and −20 dB at baseline were more likely to deteriorate by at least 7 dB. Figure 6 maps the frequency distribution of test locations reaching each endpoint. Most of the progressing locations are distributed within the superior and inferior arcuate regions, as well as the “nasal step” region. Therefore, when using Endpoint A, the required *pre-selected* visual field locations should be within or adjacent to a scotoma detected at baseline (i.e. study entry), preferably within the superior and inferior arcuate and the “nasal step” regions.

## Discussion

In this retrospective, longitudinal study assessing real-world data of glaucomatous patients, we found that eyes experiencing rapid 24-2 visual field MD slopes over a 2-year period were more likely to reach one of the FDA accepted endpoints during or soon after that period. These findings suggest that enriching clinical trials with a sample of patients with progressing disease could enable the detection of clinically significant, regulatory compliant endpoints in glaucoma neuroprotection trials over a relatively short period of time.

The advantages of using MD slopes for reducing the sample size requirements in glaucoma trials have been recently discussed^15–19^. Wu et al. have shown that the feasibility of glaucoma clinical trials could be improved by evaluating differences in the rate of visual field change (slopes) between randomization groups^19^. For instance, assuming a between-group treatment effect of 30%, and a 90% statistical power, 1924 participants would be required per group using more conventional event-based analysis, whereas 277 participants per group would be needed using trend analysis of the MD^19^. Proudfoot et al. reported that for an 80% power to detect between-group differences in the rate of MD change could be attained with total follow-up between 18 months and 2 years and fewer than 300 total participants^18^.

One main challenge facing neuroprotection clinical trials is the fact that all patients in such trials would be receiving some type of traditional IOP-lowering (i.e. standard care), which would increase the sample size and study duration requirements to detect a meaningful number of patients reaching a visual field endpoint^11^. In the United Kingdom Glaucoma Treatment Study (UKGTS), for instance, about 23% of patients treated with a prostaglandin reached an event-based endpoint in 2 years^6^. Nonetheless, that endpoint was a modification of the commercially available Glaucoma Progression Analysis (GPA) of the Humphrey Field Analyzer (HFA, Carl Zeiss Meditec, Inc., Dublin, CA, USA) and required three test locations to progress and be confirmed upon repeat testing. Both endpoints recommended by the FDA require at least five test locations, which would yield an even smaller number of eyes reaching an endpoint. Therefore, instead of an untreated placebo group (from an IOP standpoint), future trials need to consider enriching their population with patients likely to progress during the trial. There are many potential strategies for sample enrichment in glaucoma studies, such as using historical visual field or optical coherence tomography data, risk calculators, or excluding patients with low IOP and stable disease, among others. Regardless of the strategy chosen, which warrants further studies to assess feasibility and efficacy, the ultimate goal is to increase the number of patients reaching any given endpoint during the trial and thus increase the absolute effect size, which ultimately would reduce the sample size requirements. Our data suggests that if one such strategy enabled the enrollment of a sample progressing, on average, more rapidly than the mean MD slope of a general treated glaucoma population (−0.5 dB/year)^7^, around 50% of patients would reach an FDA recommended endpoint before 2 years from baseline, and about 80% in less than 4 years. Moreover, the performance of MD slopes to differentiate eyes reaching vs. not reaching one of the endpoints was close to 90%. This translates into a significant reduction in sample size requirements. Additionally, the advantages of employing trend analysis of the visual field MD instead of pointwise event-based endpoints ^19^ (discussed above) may further help reduce the costs of neuroprotection trials. Although a cut-off MD slope of −1.0 dB/year can help even further, the difference in predictive ability was minimal compared to −0.5 dB/year. This happened for two reasons: (1) there are far fewer patients progressing faster than −1.0 dB/year, and (2) all those patients progressing faster than −1.0 dB/year are also included in the group progressing faster than −0.5 dB/year. Finally, there are ethical issues in designing a trial where patients are losing visual field sensitivity at such rapid speed; they would likely require significant escalation of treatment with IOP lowering therapies during the trial, which could be a confounder in the analysis.

Some of the limitations of our study are its retrospective nature and the use of visual field data alone, without other clinical correlates. Nevertheless, this sample is drawn from a real-world population which is key for the translation to clinical practice. Despite the lack of other clinical data, all patients had or were suspected of having optic nerve damage and had confirmed glaucomatous visual field loss. In addition, masked review of the visual field data maintained data quality and mitigated the effect of non-glaucomatous effects on perimetric progression. As in previous clinical trials, future neuroprotection trials will also require masked reading centers ensuring the quality of the examinations and ruling out endpoints being met due to other causes. In the Ocular Hypertension Treatment Study (OHTS), for instance, it was shown that despite using an objective visual field endpoint, the role of a reading center was key to ensure the statistical power of the trial, without which the study would not have reached statistical significance^20^. Moreover, visual field tests using SITA Standard and SITA Fast were combined in the present analysis. Although there can be some improvement in pointwise sensitivities when switching from Standard to Fast algorithms, studies suggest this effect in minimal and may not influence the calculation of rates of MD change^21,22^. Finally, the frequency of testing in our sample (about two tests per year) is likely less than what a clinical trial should perform. Studies have shown that more frequent testing coupled with clustering at the beginning and end of the trial may further help improve the statistical power and reduce sample size requirements in glaucoma trials employing visual field tests and endpoint^16,23,24^.

In summary, we found that neuroprotection trials employing visual field MD slopes could meet the FDA requirements for an alternative functional progression endpoint. The MD slopes had high predictability of *both current and future* progression based upon currently accepted endpoints. Such trials would require sample enrichment with patients progressing more rapidly than the average of the treated glaucoma population. This approach would allow for shorter duration trials with a significantly smaller sample size than those used in glaucoma trials looking at functional endpoints to date and help accelerate approval of novel therapies.

## Methods

The Human Research Protection Office and Institutional Review Board at Columbia University approved the creation of the de-identified database of visual field tests included in this study. All methods were performed in accordance with the relevant guidelines and regulations of the Institutional Review Board. The study adheres to the tenets of the Declaration of Helsinki. Written informed consent was waived by the Human Research Protection Office and Institutional Review Board at Columbia University given the retrospective nature of the study.

Visual field tests were selected from the Edward S. Harkness Eye Institute at the Department of Ophthalmology at Columbia University Irving Medical Center. For this study, only the visual field data of patients with or suspected glaucoma (based on optic disc examination) were included. Only the visual field data, without their clinical correlates, were available for this project. Patients in the dataset were treated at the discretion of attending physicians.

### Inclusion and exclusion criteria

Eyes with 24-2 visual field tests performed using the Swedish Interactive Thresholding Algorithm (SITA; Standard and Fast) with white-on-white stimuli of size III were selected for the present analyses. This initial dataset included 204,781 visual field tests from 34,362 patients with or suspected glaucoma based on the attending physicians’ assessment.

The following reliability criteria were applied to the visual field tests included in this study: less than 33% false negative responses, less than 20% false positive errors, and less than 33% fixation losses^25^. To minimize the learning effect on the calculation of the slopes, the first two tests from each eye were excluded from the analyses. To select patients with glaucomatous visual field loss, only eyes with a Glaucoma Hemifield Test (GHT) “Outside Normal Limits” or Pattern Standard Deviation (PSD) probability less than 0.05 on at least two consecutive visits were included^2^. The first timepoint during the sequence of visual field tests when those criteria were met was defined as the baseline exam. All tests prior to the baseline exam were excluded from the analyses. Only eyes with baseline 24-2 MD between −3 and −15 dB were included. This range was chosen to minimize the chances of including eyes without established visual field loss as well as to mitigate the effects of severe functional loss on perimetric variability and the floor effect.

From the above, only eyes with at least seven reliable visual field tests were selected, of which the first five or more tests had to be done within 2 years. This sequence is herein called Sequence 1. This criterion was chosen to simulate a 2-year clinical trial while still taking into account that a minimum of five tests are needed for a reasonable calculation of slopes using linear regression. A minimum of two tests after that sequence would enable assessing changes from baseline in a follow-up examination and confirmation on a confirmatory test. This sequence after Sequence 1 is herein called Sequence 2. There were no constraints on the time between Sequences 1 and 2 given the current recommendations do not impose a time limit on the predictability of *either current or future endpoint* assessment*.*

### Endpoint analyses

To determine if the eye reached one of the recommended functional endpoints, the average of the first two tests (from Sequence 1) was used to define the pointwise baseline sensitivities. The total deviation (TD) values were used for these analyses because the current recommendations are based upon the GCP method (Carl Zeiss Meditec, Inc, Dublin, California, USA), which employs the TD instead of the pattern deviation (PD) plots for progression determination^26^. Of note, the GCP algorithm is no longer commercially available, as it has been replaced by the Glaucoma Progression Analysis (GPA), which is based upon the PD plots^27^. For consistency with the FDA’s recommendations, we kept the analyses based upon the GCP in this study. The regression models to derive the GCP’s limits of test–retest variability are adapted from the work of Heijl et al.^28^.

For Endpoint A, that is, five or more locations worsening by at least 7 dB, if at any point in time the TD sensitivity decreased below that value and later confirmed in the next follow-up test, that *test location* was deemed progressing. If at least five locations met the above criteria on the same date, the *eye* was deemed progressing at that point in time. As per the agency’s recommendation, these five test locations need to be pre-defined at baseline. Given the challenges in predicting which test locations are going to deteriorate over time, we performed a post-hoc analysis identifying the characteristics of the locations more likely to progress based on that definition.

In a similar fashion, for Endpoint B, that is, at least 5 test locations based upon the GCP algorithm, if at any point in time the TD sensitivity decreased below the GCP’s lower limit of test–retest variability and was confirmed in the next follow-up test, that *test location* was deemed progressing. If at least five locations met the above criteria on the same date, the *eye* was deemed progressing at that point in time. No constraints on the distribution of the progressing locations were required so as to resemble the GCP algorithm, that is, the five or more locations could be anywhere in the visual field.

Given the possibility that visual field changes due to non-glaucomatous causes could have detrimentally affected the determination of event-based progression ^26^ as well as the calculation of MD slopes^29^, a quality-check of all visual field sequences of the final dataset was performed by a glaucoma expert (CGDM) masked to the MD slopes and endpoint determination. If any non-glaucomatous changes in visual function were suspected (e.g., learning effect, fatigue, diffuse loss from cataract or retinal diseases), the data were excluded from the analysis.

### Statistical analysis

The visual field MD slopes of Sequence 1 were calculated using least squares linear regression, which is the statistical method most widely employed in commercially available machines. The coefficient (β) of the model: *MD (in dB)* = *intercept* + *β *×* Time (in years)* represents the slope (or speed of progression) in dB/year.

Kaplan–Meier survival curves were used to describe the cumulative survival function of eyes reaching either endpoint criterion. For these curves, data were included from Sequence 1 and for a duration of 10 years (or 8 years after Sequence 1). The date of the first exam (of at least two) in which the eye reached one of the endpoints was used to define time to event. We investigated two MD slope cut-offs and their predictive value: (i) faster than −0.5 dB/year, i.e., describing eyes progressing more rapidly than the average progression rate in treated glaucoma patients^7^; and (ii) faster than −1.0 dB/year, i.e., describing eyes experiencing very rapid visual field progression^30^.

Cox Proportional Hazards models were employed to test for the statistical significance between these MD slope cut-offs and the cumulative probability of reaching either endpoint, as well as to test for the magnitude of the associations based on Hazard Ratios. The length of time from the baseline exam until 50% of eyes reached one of the endpoints (i.e., median survival probability) was assessed.

The area under the curve of Receiver Operating Characteristics curves (aucROC) was calculated to assess the performance of MD slopes in predicting Endpoints A and B. auROC value of 50% suggest no discriminatory ability, whereas values greater than 80% suggest strong performance. Linear mixed effects models were used to compare the MD slopes between eyes reaching vs. not reaching one of the endpoints. All analyses were adjusted for inter-eye correlations due to the inclusion of both eyes of the same subject using robust cluster estimation or hierarchical mixed effects models where applicable.

Computerized statistical analyses were performed with SAS version 9.4 (SAS Institute, Cary NC), Stata version 14.2 (StataCorp, College Station, TX), and R (R Foundation for Statistical Computing, Vienna, Austria). Statistical significance was defined at P < 5%.

## Data Availability

The datasets generated and/or analyzed during the current study are not publicly available due IRB guidance on data sharing but could be available from the corresponding author on reasonable request.

## Abbreviations

IOP: Intraocular pressure
MD: Mean deviation
SAP: Standard automated perimetry
GHT: Glaucoma hemifield test
PSD: Pattern standard deviation
SD: Standard deviation
OHTS: Ocular Hypertension Treatment Study
UKTGS: United Kingdom Glaucoma Treatment Study
